# Peptides of Matrix Gla Protein Inhibit Nucleation and Growth of Hydroxyapatite and Calcium Oxalate Monohydrate Crystals

**DOI:** 10.1371/journal.pone.0080344

**Published:** 2013-11-12

**Authors:** Maria Goiko, Joshua Dierolf, Jared S. Gleberzon, Yinyin Liao, Bernd Grohe, Harvey A. Goldberg, John R. de Bruyn, Graeme K. Hunter

**Affiliations:** 1 Department of Physics & Astronomy, University of Western Ontario, London, Canada; 2 School of Dentistry, University of Western Ontario, London, Canada; 3 Department of Biochemistry, University of Western Ontario, London, Canada; INSERM, France

## Abstract

Matrix Gla protein (MGP) is a phosphorylated and γ-carboxylated protein that has been shown to prevent the deposition of hydroxyapatite crystals in the walls of blood vessels. MGP is also expressed in kidney and may inhibit the formation of kidney stones, which mainly consist of another crystalline phase, calcium oxalate monohydrate. To determine the mechanism by which MGP prevents soft-tissue calcification, we have synthesized peptides corresponding to the phosphorylated and γ-carboxylated sequences of human MGP in both post-translationally modified and non-modified forms. The effects of these peptides on hydroxyapatite formation and calcium oxalate crystallization were quantified using dynamic light scattering and scanning electron microscopy, respectively. Peptides YGlapS (MGP1-14: YγEpSHEpSMEpSYELNP), YEpS (YEpSHEpSMEpSYELNP), YGlaS (YγESHESMESYELNP) and SK-Gla (MGP43-56: SKPVHγELNRγEACDD) inhibited formation of hydroxyapatite in order of potency YGlapS > YEpS > YGlaS > SK-Gla. The effects of YGlapS, YEpS and YGlaS on hydroxyapatite formation were on both crystal nucleation and growth; the effect of SK-Gla was on nucleation. YGlapS and YEpS significantly inhibited the growth of calcium oxalate monohydrate crystals, while simultaneously promoting the formation of calcium oxalate dihydrate. The effects of these phosphopeptides on calcium oxalate monohydrate formation were on growth of crystals rather than nucleation. We have shown that the use of dynamic light scattering allows inhibitors of hydroxyapatite nucleation and growth to be distinguished. We have also demonstrated for the first time that MGP peptides inhibit the formation of calcium oxalate monohydrate. Based on the latter finding, we propose that MGP function not only to prevent blood-vessel calcification but also to inhibit stone formation in kidney.

## Introduction

 Matrix gla protein (MGP) is a phosphorylated and γ-carboxylated protein expressed at high levels in heart, lung and kidney [1]. MGP is a highly conserved 84-amino acid protein that contains 5 residues of γ-carboxyglutamic acid (gla): one at amino acid 2 and the rest in the centre of the molecule (amino acids 37, 41, 48 and 52) [2]. In addition, there are 3 sites of serine phosphorylation near the N-terminus (amino acids 3, 6 and 9) [3]. The C-terminal third of MGP is quite hydrophobic, and consequently the protein is poorly soluble [2,4,5] . Apparently for this reason, very little is known about MGP’s structure, although one study reported that synthetic MGP (γ-carboxylated but not phosphorylated) has ~21% α-helix [5].

 Mice lacking the *Mgp* gene exhibit massive calcification of the medial layer of blood vessels and die from arterial rupture soon after birth [6]. A similar pattern of calcification is seen when rats are given the vitamin K antagonist warfarin, which inhibits γ-carboxylation, implicating the gla residues of MGP in the anticalcification function of the protein [7]. In humans, expression of the *Mgp* gene is upregulated in human atherosclerotic plaque [8], suggesting that the protein is an inducible inhibitor of calcification. Although undercarboxylation of MGP is associated with aortic stenosis [9], warfarin treatment does not cause a significant increase in coronary artery calcification [10]. Nonetheless, it appears clear that MGP functions as an inhibitor of blood-vessel calcification, and that the gla residues play an important role in this process. 

 To further investigate the role of post-translational modifications in the anti-calcification activity of MGP, Schurgers et al. studied cultures of vascular smooth muscle cells. In media containing high concentrations of calcium and/or phosphate, these cultures produce a calcified matrix; like atherosclerotic plaque, the mineral phase is hydroxyapatite (HA). Addition of synthetic peptides corresponding to full-length MGP, amino acids 35-54 or amino acids 3-15 inhibited calcification of vascular smooth muscle cells at a concentration of 200 nM, while non-post-translationally modified versions of these molecules had no significant effects [11]. 

 The above-mentioned studies did not address the mechanism by which MGP inhibits calcification. We hypothesized that MGP adsorbs to HA and inhibits further growth of the crystals, which has previously been shown for other calcification-inhibiting proteins [12]. To test this hypothesis, we used a combination of simulation and experimentation. For simulation of the adsorption of MGP to HA, the 84-residue human MGP sequence was divided into six 14-amino-acid virtual peptides: YGlapS, FIN, QR-Gla, SK-Gla, YRL and AAY. The interaction of each sequence with the {100} and {001} faces of HA studied by molecular dynamics. Peptide YGlapS contains one gla and 3 phosphoserines, and was therefore also simulated in non-γ-carboxylated (YEpS), non-phosphorylated (YGlaS) and non-γ-carboxylated/non-phosphorylated (YES) forms. QR-Gla and SK-Gla each contain two glas, and therefore were also synthesized in non-γ-carboxylated forms (QR-E and SK-E, respectively). In the experimental arm of the study, synthetic peptides corresponding to each of the virtual peptides were synthesized. Inhibition of HA growth by the synthetic MGP peptides was quantified by the constant-composition/seeded growth method [13]. Results from simulation and experimentation were in excellent agreement. Peptides YGlapS (YγEpSHEpSMEpSYELNP) and SK-Gla (SKPVHγELNRγEACDD) adsorbed most strongly to HA and were also potent inhibitors of HA growth (IC_50_ values of 1.48 and 2.92 μM, respectively). The adsorption and inhibitory activities of YGlapS were dependent upon phosphorylation but not γ-carboxylation, whereas those of SK-Gla were dependent upon γ-carboxylation [14]. These findings suggest that MGP inhibits arterial calcification by adsorbing to and inhibiting the growth of HA crystals, and that this activity involves the phosphorylated N-terminus and central gla-containing region of the protein. 

 MGP is expressed at high levels in kidney [1,15,16] and its expression is upregulated in animal models of kidney-stone disease [17,18]. In addition, a polymorphism of the *Mgp* gene has been linked to stone disease in Japanese [19] and Chinese [20] populations. This raises the possibility that MGP plays a role in preventing the ectopic calcification of kidney. Unlike atherosclerotic plaque, kidney stones contain a variety of mineral phases. While calcium oxalates (CaOx) are the most abundant, HA and other calcium phosphates are also often present in stones [21,22]. 

 Crystal formation involves two processes: nucleation and growth. In the present study, we have examined the effects of synthetic MGP peptides on the *in vitro* formation of HA by dynamic light scattering (DLS), which, unlike the constant-composition/seeded growth method used in our previous study [14], allowed us to examine the effects of these peptides on crystal nucleation. Because CaOx crystals tend to nucleate on surfaces rather than in solution, DLS cannot be used. Instead, we employed scanning electron microscopy to study the effects of MGP peptides on the nucleation and growth of calcium oxalate monohydrate (COM) and calcium oxalate dihydrate (COD) crystals. The non-post-translationally modified MGP peptides FIN (MGP15-28), AAY (MGP57-70), and YRL (MGP71-84) were not included in the present analysis because they exhibited no adsorption to HA or inhibition of HA growth in our previous study [14]. QR-Gla (MGP29-42) and QR-E (non-γ-carboxylated MGP29-42) were studied, but had no significant effects on HA or CaOx formation. Therefore, data from these peptides are not included here. 

## Methods

### Physicochemical characterization of peptides

 MGP peptides were synthesized as previously described [14]. The physicochemical characteristics of these peptides are listed in Table 1. Isoelectric points (pI), net charges and hydrophilicity values were determined as previously described [23]. Hydrophilicity data were calculated on the basis of an empirical hydrophilicity scale for individual amino acids elaborated by Hopp and Woods [24]. Estimation of hydrophilicity data for posttranslational modifications (e.g. phosphate, γ-carboxyglutamate) were carried out as previous described [23].

**Table 1:** Chemical characteristics of MGP peptides.

Peptide	Sequence	M_W_^A^ [g/mol]	Isoelectric point ^B^	Net charge at pH 6.7 / 7.4 ^B^	Hydrophilicity [kJ/mol] ^C^
YGlapS	YγEpSHEpSMEpSYELNP	1998.8	2.23	- 8.6/- 9.1	56.5/4.03
YEpS	YEpSHEpSMEpSYELNP	1954.7	2.36	- 8.1/- 8.6	54.4/3.89
YGlaS	YγESHESMESYELNP	1758.7	3.84	- 4.1/- 4.6	22.6/1.61
YES	YESHESMESYELNP	1714.8	4.00	- 3.6/- 4.1	20.5/1.46
SK-Gla	SKPVHγELNRγEACDD	1700.8	4.52	- 2.7/- 3.2	59.4/4.24
SK-E	SKPVHELNRγEACDD	1612.8	4.89	- 1.7/- 2.2	55.2/3.94

^A^ Molecular weight (electrospray mass spectrometry); ^B^ (http://ca.expasy.org, different tools); ^C^ Data are given for the complete molecule and the amino acid average within that molecule, calculated on basis of the empirical hydrophilicity scale of Hopp and Woods [24] and by estimation of hydrophilicity data for posttranslational modifications [23].

### Circular dichroism spectropolarimetry

 Circular dichroism spectra were recorded using a Jasco J-810 spectropolarimeter connected to a Peltier temperature-control system. Peptides were dissolved at a concentration of 0.22 mg/mL in distilled water. Scans were measured from 200 nm to 260 nm. Each peptide solution was scanned at 37°C in a cell with a path length of 1 mm, with a scan speed of 100 nm/min and step size of 0.5 nm. Solutions were scanned 10 times and the resulting spectra were averaged. After subtraction of blank (water) scans, peptide raw data were converted to mean residue ellipticity (θ) in units of degree cm^2^ dmol^-1^ by standard procedures. CONTINLL, CDSSTR and SELCON3 algorithms were used to estimate peptide secondary structure from the UV CD spectra generated with the protein reference set "SMP50." Estimates from the three algorithms were then averaged using Prism 4 (GraphPad).

### Hydroxyapatite growth

 Three working solutions were prepared daily as described in [25], composed of 24 mM CaCl_2_, 150 mM NaCl and 18.75 mM Tris (calcium solution); 15 mM Na_2_HPO_4_, 150 mM NaCl and 18.75 mM Tris (phosphate solution); and 150 mM NaCl and 18.75 mM Tris (Tris solution). For each experimental run, measured volumes of the calcium and phosphate solutions were added to Tris solution containing the desired concentration of peptide (0-10 μg/ml) in a 5-ml borosilicate glass tube, giving a final volume of 1.8 ml. The ratio of Ca^2+^ and PO_4_^3-^ concentrations in the resultant solution was fixed at 1.60. The concentrations of Ca^2+^ and PO_4_^3-^ were chosen to give a reasonable rate of crystal formation (intensity of ~100 kHz by the end of the experiment) in control runs with no added peptide. The level of supersaturation required for this varied from day to day, presumably because of minor variations in temperature, pH and presence of impurities. For YGlapS, YEpS, YGlaS and YES, [Ca] = 5.67 mM and [PO_4_] = 3.54 mM; for SK-Gla, [Ca] = 7.33 mM and [PO_4_] = 4.58 mM; for SK-E, [Ca] = 7.00 mM and [PO_4_] = 4.38 mM. All measurements for a given peptide were performed within a span of six to eight hours. The effects of various peptide concentrations on HA formation were compared to that day’s control. Solutions were prepared and experiments performed at room temperature. 

### Dynamic light scattering

 Dynamic light scattering experiments were performed using an ALV CGS-3 goniometer-based light scattering system. The procedure and data analysis were similar to those described in ref [25].. Scattering data were collected over a period of forty minutes immediately following the preparation of a crystal-forming solution. The autocorrelation function of the scattered light intensity and the mean scattered intensity *I*_m_ were automatically calculated and saved at 30-s intervals. *I*_m_ increases with both the concentration and size of the scattering particles. The electric field autocorrelation function was calculated from the measured intensity autocorrelation function using the Siegert relation [26]. The mean hydrodynamic ($\bar{r_{h}}$) of the scattering particles at each 30-s time step was determined from the decay of field autocorrelation function using the method of cumulants [27] and a standard nonlinear least-squares fitting algorithm. 

### Calcium oxalate growth

 To study effects of MGP peptides on COM and COD growth habit and volumes of precipitate, crystals were grown on freshly cleaved mica from solutions containing [Ca^2+^] = [C_2_O_4_^2-^] = 1 mM as previously described [28,29]. Under these conditions, the supersaturation (calculated as σ = 1/2 ln [*a*_Ca_·*a*_Ox_/*K*_SP_] [23]) was 2.86 for COM and 2.31 for COD using solubility products *K*_SP_ for COM (*K*_SP_;_COM_ = 2.24 · 10^-9^ M^2^) or COD (*K*_SP_;_COD_ = 6.76 · 10^-9^ M^2^) at 37°C [30]. Peptides were studied at 0-20 μg/ml.

### Scanning electron microscopy

 Scanning electron microscopy (SEM; A LEO 1540XB, Carl Zeiss, Germany) was used to study the precipitates on mica substrates without metal coating, at an acceleration voltage of 1 kV and a working distance of 3.5-4.0 mm. Using SEM micrographs, COM <001> and <010> dimensions were measured from six {100}-nucleated crystals, and <001> and <100> dimensions were measured from six {010}-nucleated crystals. Volumes were calculated as mean area of the {100} face x mean <010> dimension x number of crystals/mm^2^ [23,29]. COD dimensions were analyzed from six crystals nucleated from various orientations. Data from each crystal (e.g., edge length, angles) were entered in a calculation routine and crystal volumes determined. Overall volumes were calculated as mean crystal volume x number of crystals/mm^2^, as previous described [23,29]. Data were analyzed by one-way analysis of variance followed by Dunnett’s multiple correlations test using Prism 4 (GraphPad).

## Results

### Structure of MGP peptides

**Figure 1:**
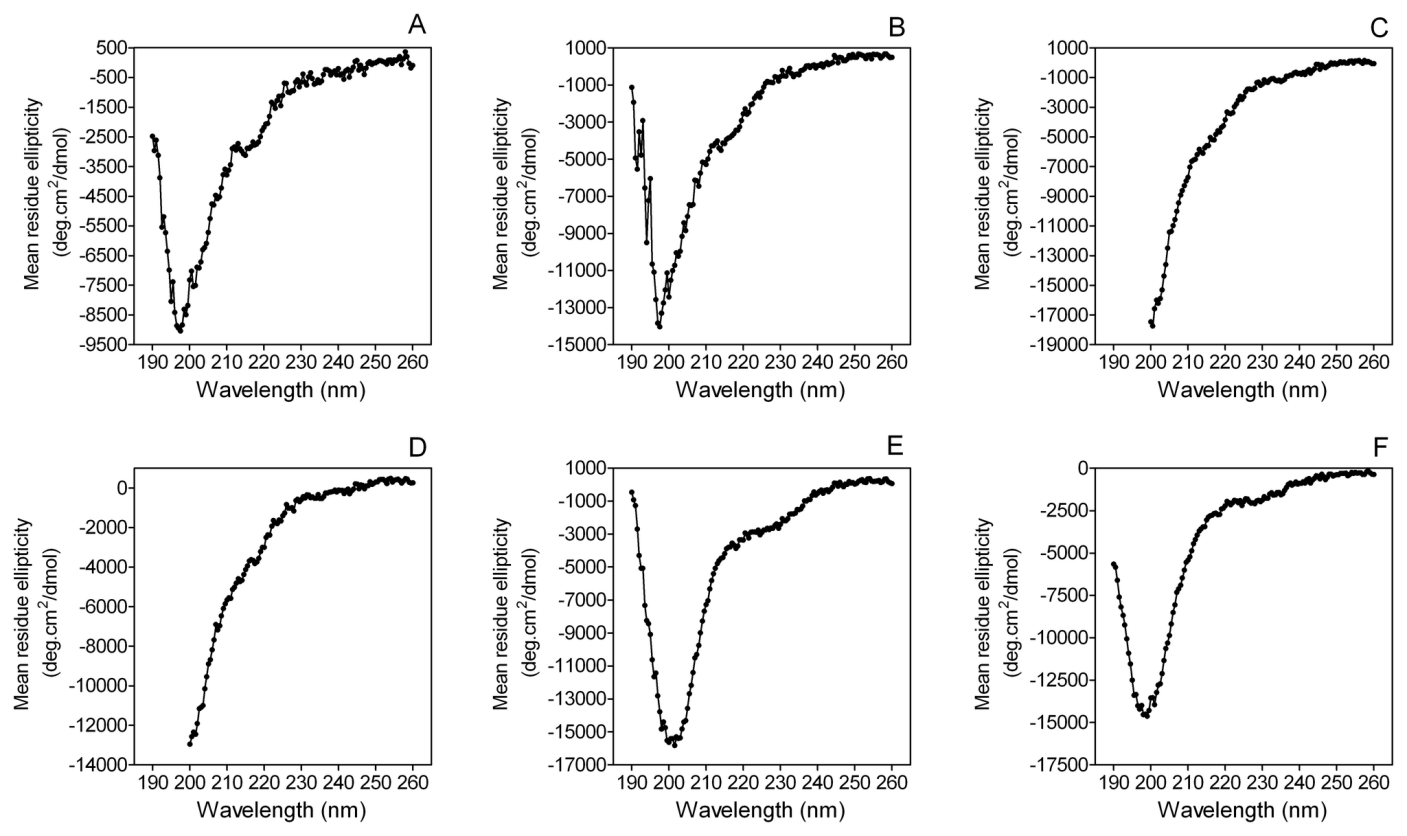
Circular dichroism spectropolarimetry analyses of MGP peptides. A. YGlapS B. YEpS C. YGlaS D. YES E. SK-Gla F. SK-E .

 The sequences and physicochemical properties of the MGP peptides used in this study are shown in Table 1. The secondary structures of these peptides were studied by circular dichroism spectropolarimetry (Figure 1). Deconvolution of the CD spectra indicated that these peptides are approximately 35% unordered with low helical content and high contents of turn and β-strand (Table 2). 

**Table 2:** Analysis of circular dichroism spectropolarimetry of MGP peptides.

Peptide	α-helix	Distorted α-helix	β-strand	Distorted β-strand	Turn	Unordered
YGlapS	0.9±0.8	5.6±1.8	23.9±0.9	13.1±0.6	20.6±1.9	30.5±4.1
YEpS	0.1±0.1	3.7±1.43	22.2±1.43	14.1±0.2	22.4±0.2	36.8±0.8
YGlaS	0.3±0.3	5.4±1.2	19.1±1.1	13.2±0.3	25.3±0.5	35.7±1.42
YES	1.1±1.0	2.2±2.1	12.8±7.8	10.8±2.1	29.2±5.7	44.1±5.7
SK-Gla	2.1±1.7	8.1±1.9	19.1±2.3	11.6±0.2	23.8±1.9	33.4±3.7
SK-E	1.9±1.2	5.7±1.6	19.1±2.0	11.8±0.1	23.4±0.3	38.7±1.2

Spectra shown in Figure 1 were analyzed with CDPro. Secondary structures percentages derived by the CONTINLL, CDSSTR and SELCON3 algorithms were averaged (± SEM).

### Effects of MGP peptides on HA nucleation and growth

**Figure 2:**
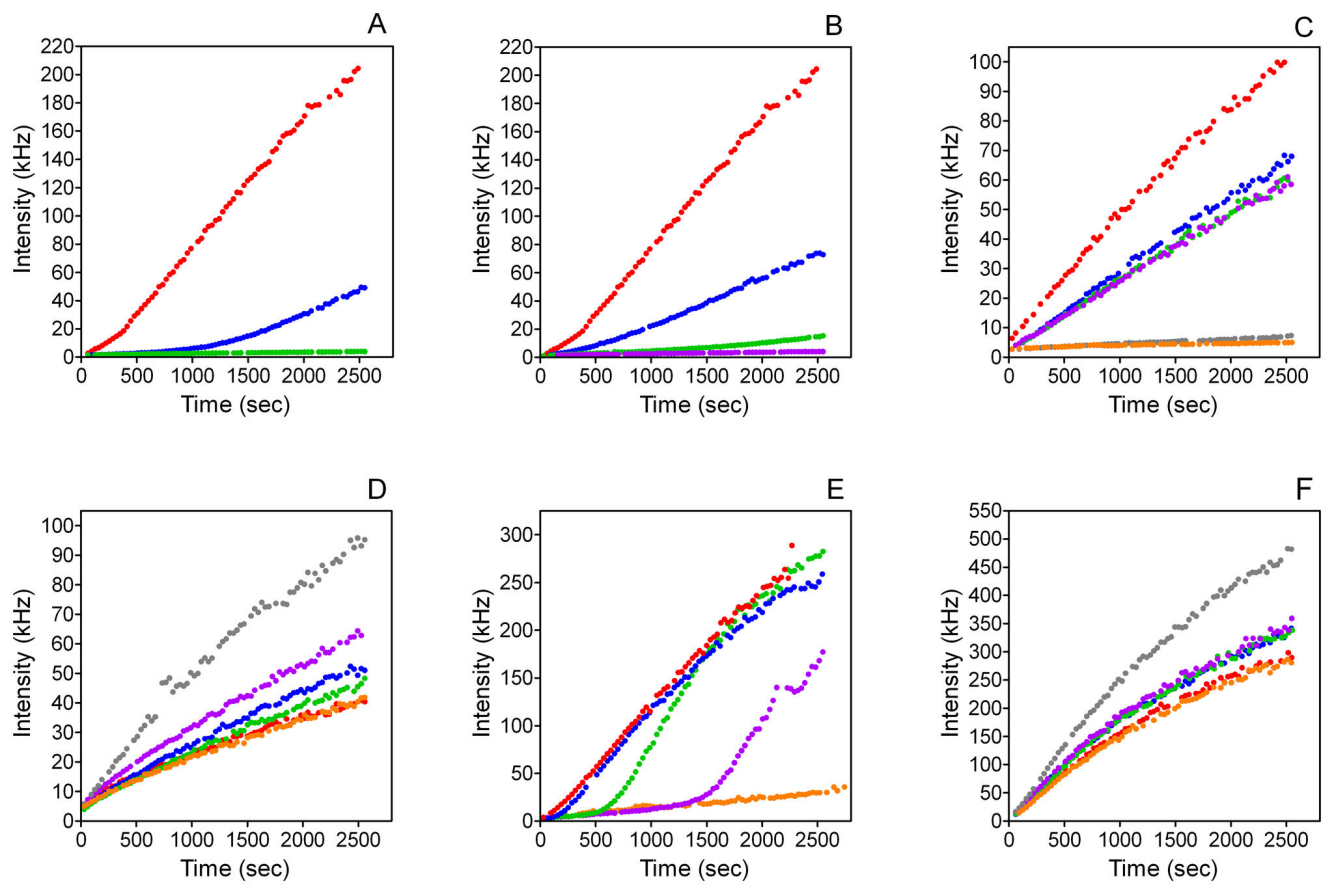
Effects of MGP peptides on intensity of dynamic light scattering. A. YGlapS . B. YEpS . C. YGlaS .

 The effects of MGP peptides on HA formation were studied by dynamic light scattering (DLS). Figure 2 shows plots of DLS intensity against time for peptide YGlapS and its variants, YGlaS, YEpS and YES, as well as SK-Gla and its variant, SK-E. DLS intensity is a function of both the size and number of scattering particles. Of the 6 peptides studied, YGlapS resulted in the greatest decreases in intensity, with the maximal effect occurring at 3 μg/ml (Figure 2A). YEpS, the non-γ-carboxylated version of this peptide, was almost as potent (Figure 2B). YGlapS, the nonphosphorylated version of YGlapS, was less potent than YEpS, with 7 μg/ml peptide required to maximally decrease the change in intensity with time (Figure 2C). YES, which is neither γ-carboxylated nor phosphorylated, resulted in only a small decrease in intensity at 10 μg/ml, whereas lower concentrations appeared to promote crystallization (Figure 2D). These findings indicate that both the 3 phosphate groups and the single γ-carboxylate group contribute to the HA-inhibiting effect of YGlapS. 

E. SK-Gla .

F. SK-E .

Red – control, blue - 1 μg/ml, green - 3 μg/ml, magenta - 5 μg/ml, grey - 7 μg/ml, orange - 10 μg/ml.

 Increasing concentrations of SK-Gla caused decreased DLS intensities (Figure 2E), although the effect was less than that of YGlapS or YEpS (Figure 2A, B), with some crystal formation occurring at 10 µg/ml. SK-E had no significant effect on intensity (Figure 2F), showing that the inhibitory effect of SK-Gla is critically dependent upon γ-carboxylation of this peptide. 

**Figure 3:**
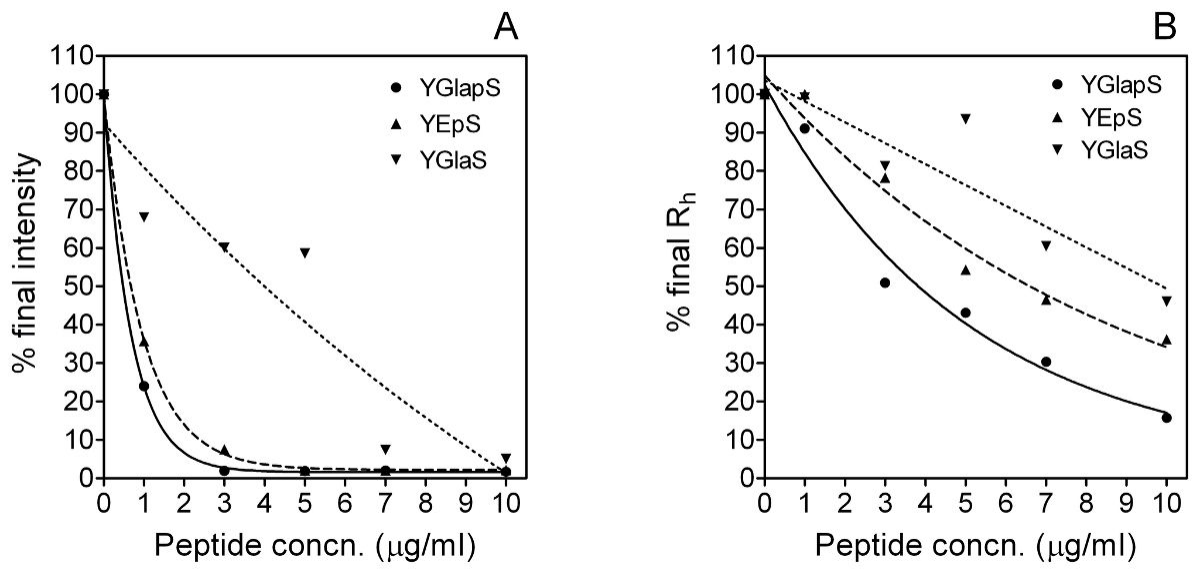
Effects of YGlapS and YEpS on HA formation as measured by dynamic light scattering. A. Intensity. B. Hydrodynamic radius. Data from Figures 2 and 4 were used. For YGlapS and YEpS, data were fitted to one-site exponential decay curves. Data for YGlaS were analyzed by linear regression.

 To compare the effects of YGlapS, YEpS and YGlaS on HA formation, intensities at the end of the incubation were plotted against peptide concentration (Figure 3A). From these plots, peptide concentrations causing half-maximal effect on intensity (IC_50_I) could be calculated. The IC_50_ *I* values for YGlapS, YEpS and YGlaS were 0.467 μg/ml (0.234 μM), 0.654 μg/ml (0.335 μM) and 4.92 μg/ml (2.80 μM), respectively. 

**Figure 4:**
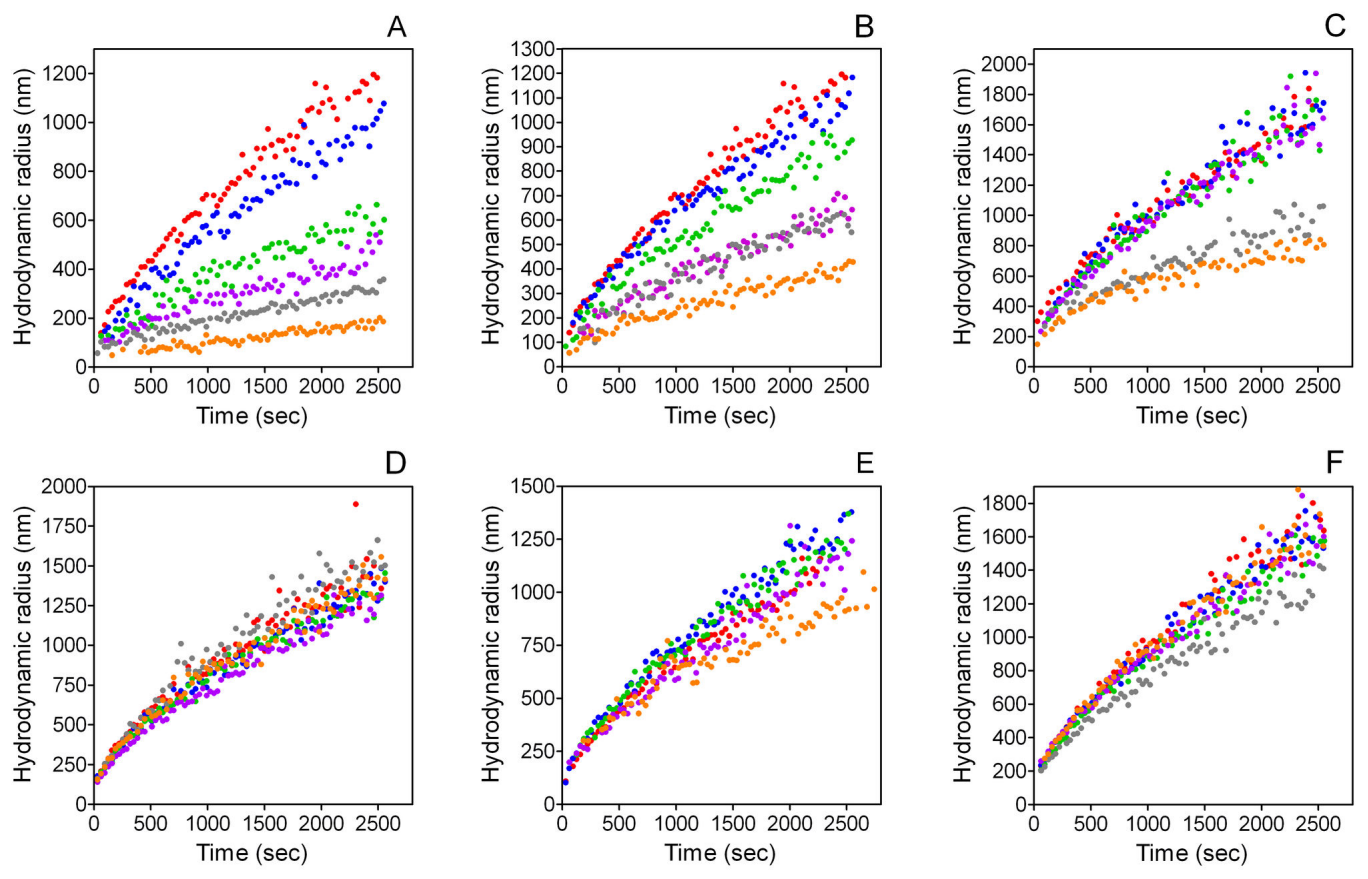
Effect of MGP peptides on hydrodynamic radii (R_h_) as measured by dynamic light scattering. A. YGlapS. B. YEpS. C. YGlaS.

 Hydrodynamic radius (R_h_) measurements are shown in Figure 4. YGlapS decreased R_h_ in a concentration-dependent manner (Figure 4A). YEpS and YGlaS also caused decreases in R_h_, although the effects are less dramatic than that of YGlapS (Figure 4B, C). YES and SK-E did not have significant effects on R_h_ (Figure 4D, F). SK-Gla slightly decreased R_h_ (Figure 4E). 

E. SK-Gla.

F. SK-E .

Red – control, blue - 1 μg/ml, green - 3 μg/ml, magenta - 5 μg/ml, grey - 7 μg/ml, orange - 10 μg/ml.

 To compare the effects of YGlapS, YEpS and YGlaS on HA growth, final R_h_ values were plotted against peptide concentration (Figure 3B). The calculated IC_50_R_h_ values for YGlapS, YEpS and YGlaS were 3.53 μg/ml (1.76 μM), 6.15 μg/ml (3.15 μM), and 9.56 μg/ml (5.44 μM), respectively. 

**Figure 5:**
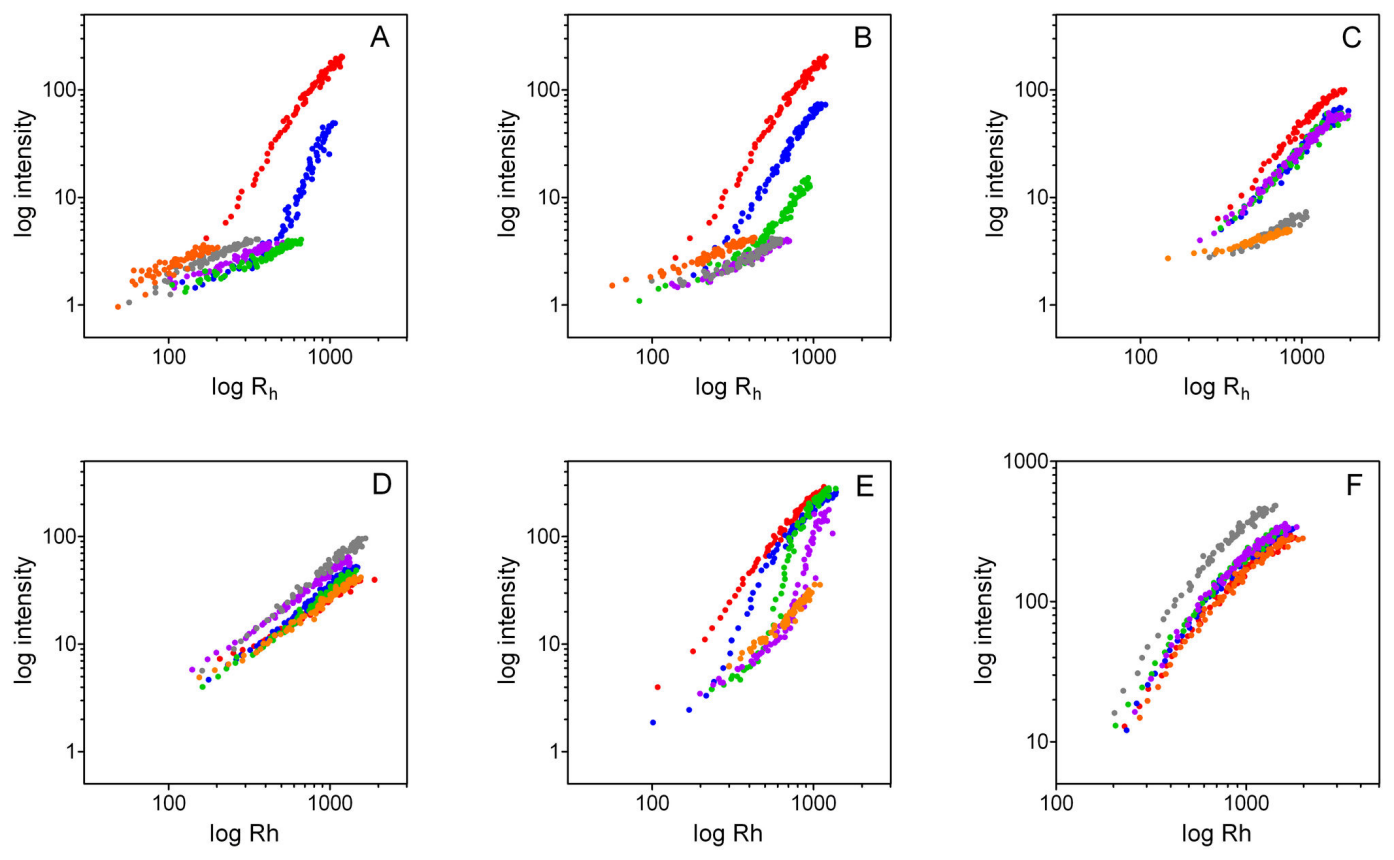
Dependence of dynamic-light-scattering intensity on hydrodynamic radius. Intensity data shown in Figure 2 were plotted against hydrodynamic radius data shown in Figure 4. A. YGlapS. B. YEpS. C. YGlaS.

 Whereas R_h_ is a well-defined measure of characteristic crystal size, intensity is a complex function of both crystal size and concentration. To determine the effects of MGP peptides on the concentration of crystals, and therefore on nucleation of HA, we plotted intensities against corresponding R_h_ values for all concentrations of each peptide (Figure 5). If a peptide causes a decrease in intensity at a given R_h_ value, this can only be due to a decreased concentration of crystals (implying inhibition of nucleation). The log-log plots shown in Figure 5 exhibit a sigmoid relationship between intensity and R_h_ for control (peptide-free) solutions. Addition of YGlapS results in less-steep gradients at all concentrations ≥3 μg/ml, showing that these concentrations inhibit HA nucleation (Figure 5A). For YEpS, concentrations ≥5 μg/ml inhibited nucleation, as shown by decreased gradients compared to control (Figure 5B). For YGlapS, inhibition of HA nucleation was only observed at peptide concentrations ≥7 μg/ml (Figure 5C). The remaining peptides – YES, SK-Gla and SK-E – had no effect on nucleation (Figure 5D, E, F). This analysis suggests that YGlapS is a more potent inhibitor of HA nucleation than YEpS, which in turn is more potent than YGlaS. 

E. SK-Gla.

F. SK-E.

Red – control, blue - 1 μg/ml, green - 3 μg/ml, magenta - 5 μg/ml, grey - 7 μg/ml, orange - 10 μg/ml.

### Effects of MGP peptides on calcium oxalate crystallization

**Figure 6:**
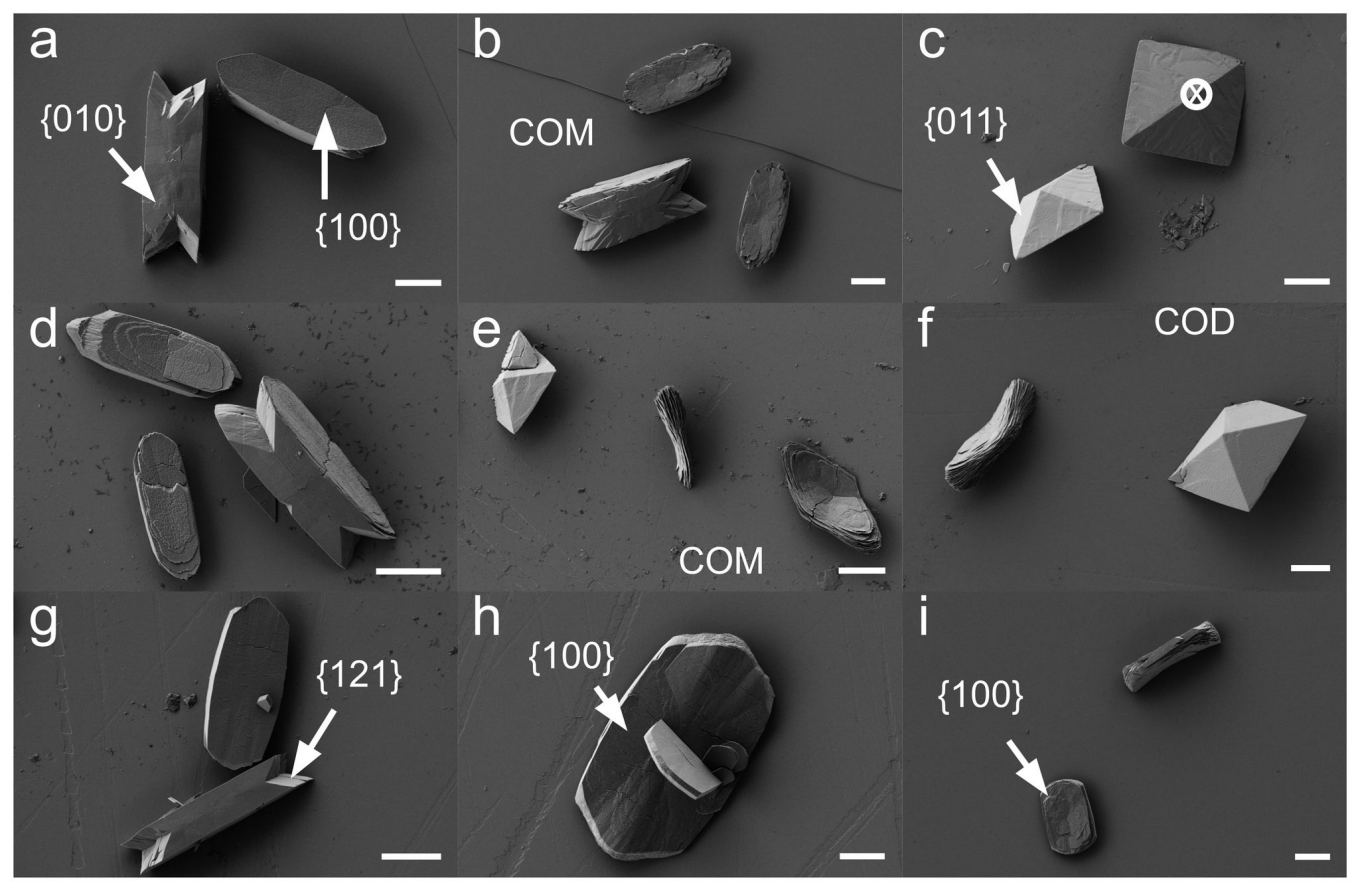
Scanning electron microscopy of CaOx crystals grown in the presence and absence of MGP peptides. A. Control (scale bar 4 μm). B. YGlapS, 1 μg/ml (scale bar 6 μm) . C. YGlapS, 5 μg/ml (cross – view parallel to <001>) (scale bar 4 μm) . D. YEpS, 1 μg/ml (scale bar 4 μm). E. YEpS, 5 μg/ml (scale bar 4 μm). F. YEpS, 20 μg/ml (scale bar 4 μm). G. SK-Gla, 5 μg/ml (scale bar 6 μm). H. SK-Gla, 10 μg/ml (scale bar 4 μm). I. SK-Gla, 20 μg/ml (scale bar 6 μm). Arrows point to the three faces developed in penetration twin COM crystals (Miller indices {100}, {010} and {121}) and the single face developed in bipyramid COD crystals (Miller index {011}) [28].

 As calcium oxalate crystals prefer to nucleate on charged surfaces rather than in solution, we were unable to use DLS for these studies. Instead, we used SEM to quantify COM and COD formed in the presence of MGP peptides at concentrations up to 20 µg/ml. From scanning electron micrographs, COM and COD crystals could readily be distinguished from one another: the former are penetration twins with monoclinic symmetry, the latter are rectangular bipyramids (Figure 6). Under control (no peptide) conditions,there was very little COD formation (Figure 6A). In the presence of higher concentrations of YGlapS or YEpS, COD crystals were commonly seen (Figure 6C, F). At lower concentrations of these phosphopeptides or at high concentrations of SK-Gla, COM crystals were smaller, with rounder interfacial edges, rough surfaces and apparent loss of twinning (Figure 6B, E, I). 

**Figure 7:**
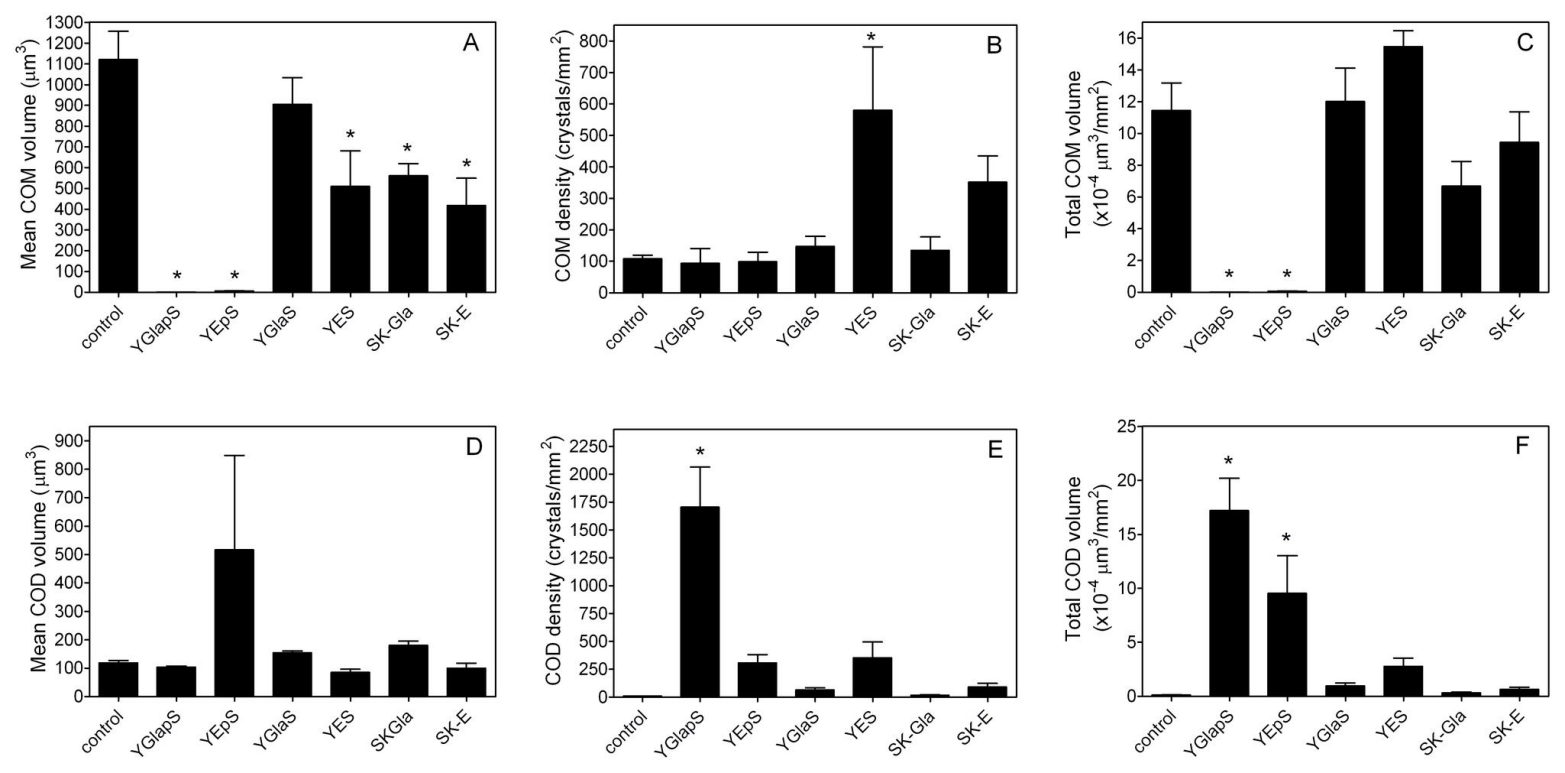
Effect of MGP peptides on calcium oxalate crystallization as measured by scanning electron microscopy. All peptides were used at 20 μg/ml. A. Mean COM crystal volume. B. COM crystal density. C. Total COM volume (A x B). D. Mean COD crystal volume. E. COD crystal density. F. Total COD volume (D x E). Error bars represent standard error of themean. *Significantly different from control, P<0.01.

 To quantify these effects, mean crystal volumes (μm^3^) and mean crystal densities (crystals/mm^2^) were measured for each crystal polymorph. The mean volumes and densities were then combined to give total volumes of COM and COD (μm^3^/mm^2^). Data obtained at 20 μg/ml peptide are shown in Figure 7. Comparison of panels C and F shows that, in the absence of peptide, formation of COM is greatly favored over COD under the conditions used. 

 COM data are shown in the upper panels of Figure 7. All peptides except YGlaS significantly decreased the mean size of COM crystals, although the phosphopeptides YGlapS and YEpS had by far the most inhibitory effects (Figure 7A). YES, which increased the number of COM crystals formed, was the only peptide to significantly affect this value (Figure 7B). Combining mean crystal sizes and densities showed that only YGlapS and YEpS significantly decreased total COM volumes (Figure 7C). 

 COD data are shown in the lower panels of Figure 7. None of the peptides caused a significant effect on mean COD volume (Figure 7D). Only YGlapS significantly increased the density of COD crystals (Figure 7E). Total volumes of COD were significantly increased by both YGlapS and YEpS (Figure 7F). 

**Figure 8:**
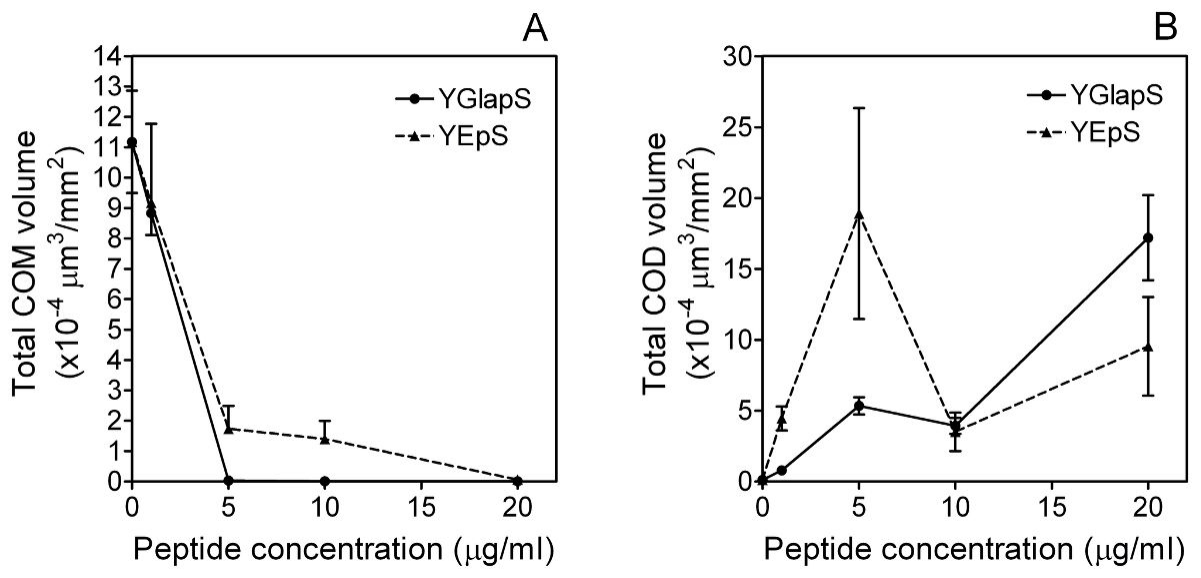
Effects of YGlapS and YEpS on COM (A) and COD (B) formation as measured by scanning electron microscopy. Error bars represent standard error of the mean.

 The effects of peptide concentration on calcium oxalate crystallization are shown in Figure 8. YGlapS exhibited almost complete inhibition of COM formation at 1 μg/ml, whereas YEpS only achieved a comparable level of inhibition at a concentration of 20 μg/ml (Figure 8A). The effect of the phosphopeptides on COD formation appears to be biphasic, with higher COD volumes seen at a YGlapS or YEpS concentration of 5 μg/ml than at 10 μg/ml (Figure 8B). 

## Discussion

 Most mineral-binding proteins are disordered; in fact, these proteins have been claimed to be the most disordered class of proteins [31]. We recently suggested that this lack of order assists proteins in interacting with mineral crystals, by facilitating sequential formation of ionic bonds [32]. However, some mineral-binding proteins are well-folded. Osteocalcin, a gla-containing protein that is essentially restricted to mineralized tissues, has been shown to adsorb to HA and inhibit HA formation [33,34]. By NMR spectroscopy [35] and X-ray diffraction [36], it was shown that, while OC is mostly α-helical, its N-terminus is disordered. Interestingly, the 3 gla residues are all within a single helical segment and project from the same face of the helix. 

 CD analysis of the MGP peptides used in the present study revealed an almost complete absence of α-helix. It may be that the 14-mer peptides are too short to fold independently. An analysis of MGP using PONDR (Predictor of Naturally Disordered Proteins) suggested that the sequences corresponding to YGlapS and SK-Gla are disordered, but most of the remainder of the molecule is ordered (unpublished). 

The effects of these peptides on HA formation were studied by DLS. DLS intensities are a function of both the concentration and size of scattering particles. The average hydrodynamic radius (R_h_) of the scattering particles can be calculated from the decay rate of the autocorrelation function of the scattered intensity. It is not feasible to measure the concentration of scatters directly. If the scattering particles are crystals in a supersaturated solution, a time-dependent increase in DLS intensity can be due to either nucleation (increased concentration of crystals) or growth (increased size of crystals). As the change in R_h_ over time is a measure of crystal growth, we were able to determine the effects of MGP peptides on HA growth. To gain insight into the effects of MGP peptides on crystal nucleation, we plotted intensity against R_h_ over a range of peptide concentrations. If a peptide reduces the intensity of light scattered over a range of hydrodynamic radii, this can only due to a reduction in the concentration of scattering particles – i.e., inhibition of crystal nucleation. By these means, we were able, for the first time, to study the effects of MGP peptides on HA nucleation. 

According to the above definitions of crystal formation, nucleation and growth, 4 of the 6 MGP peptides studied inhibit HA formation, in the order YGlapS > YEpS > YGlaS > SK-Gla, with YES and SK-E having no effect. The same four peptides inhibited HA growth, in the same order of potency, with YES and SK-E again having no inhibitory effect. Three of the peptides inhibited HA nucleation, in the order YGlapS > YEpS > YGlaS, with YES, SK-Gla and SK-E having no effect.

 Based on these findings we conclude that, in general, MGP peptides that inhibit HA growth also inhibit nucleation of HA. The exception was SK-Gla, which had a weak effect on nucleation and none on growth.

 Comparisons between post-translationally modified and non-post-translationally versions of the same peptide allow us to deduce the relative importances of phosphate and γ-carboxylate groups in the inhibition of HA formation. In terms of DLS intensity, YGlapS was more potent (IC_50_I = 0.234 μM) than YEpS (IC_50_I = 0.335 μM), which in turn was more potent than YGlaS (IC_50_I = 2.80 μM). In terms of R_h_, the IC_50_ values measured for YGlapS, YEpS and YGlaS were 1.76, 3.15 and 5.44 μM, respectively. Although we were not able to quantify the effects of these peptides on HA nucleation, it is clear that the same order of potency obtains. These analyses show that, although both phosphate and γ-carboxylate groups contribute to the HA-inhibiting activity of the YGlapS peptide, which corresponds to amino acids 1-14 of human MGP, phosphate groups are more important. This is not surprising, as the peptide has 3 phosphoserines and only one gla. 

 In general, it appears that the role of protein anionic groups in adsorption to biomineral crystals is simply to contribute to the net negative charge of the protein [23,37]. Note that the order of potency of MGP peptides in inhibiting HA nucleation and growth corresponds exactly to order of peptide negative charge (Table 1). There is no compelling reason to believe that there is any specificity with respect to the chemical nature of the charged group, only with respect to its net charge.

 A novel aspect of the present study is that the effects of MGP peptides on CaOx formation were analyzed. Only two peptides, YGlapS and YEpS, caused a significant decrease in the total volume of COM. This was entirely due to inhibition of COM growth, as the number of crystals nucleated was not affected by these peptides. As described above, YGlapS and YEpS are also the most potent inhibitor of HA formation as measured by DLS. In that case, however, the effect is on crystal nucleation and growth. 

Another difference between the effects of MGP peptides on HA and calcium oxalate crystallization is that, in the latter case, inhibition of COM formation by YGlapS or YEpS is associated with concomitant formation of COD – a CaOx polymorph that is not normally favoured under the conditions used. From our measurements of COD density and average volume, it is clear that the effect of YGlapS is entirely on nucleation, while the effect of YEpS appears to be on both nucleation and growth (although neither is significantly increased). Inhibition of COM and promotion of COD are also mediated by highly anionic peptides of the mineral-binding protein osteopontin [29,38]. In that case, and presumably in the present one, nucleation of specific COD faces by peptide adsorbed to the mica substrate appears to be responsible. 

This is the first study showing that MGP inhibits calcium oxalate crystallization. We suggest that MGP, like osteopontin and Tamm-Horsfall protein, plays a role in preventing stone formation in kidney. MGP is quite highly expressed in kidney [1] but has not been reported in the urine [39,40]. Therefore, as in blood vessels, this protein is probably confined to the extracellular matrix of the nephron. We therefore speculate that MGP acts to inhibit crystal formation in the subepithelial connective tissue, whereas osteopontin and Tamm-Horsfall protein prevent crystallization in the urine. 

 In conclusion, we have shown that highly anionic MGP peptides, particularly the N-terminal peptide YGlapS and its non-γ-carboxylated counterpart YEpS, inhibit the formation of two biominerals implicated in ectopic calcification, HA and COM. By use of novel approaches, we have been able to distinguish between effects on crystal nucleation and crystal growth. In the case of HA, nucleation and growth are both inhibited by MGP peptides. In the case of COM, only growth is inhibited; this is associated with the concomitant formation of COD, a less-stable polymorph of CaOx. 
